# CircRNA Role and circRNA-Dependent Network (ceRNET) in Asthenozoospermia

**DOI:** 10.3389/fendo.2020.00395

**Published:** 2020-07-10

**Authors:** Francesco Manfrevola, Teresa Chioccarelli, Gilda Cobellis, Silvia Fasano, Bruno Ferraro, Carolina Sellitto, Giovanni Marella, Riccardo Pierantoni, Rosanna Chianese

**Affiliations:** ^1^Dipartimento di Medicina Sperimentale, Università degli Studi della Campania Luigi Vanvitelli, Naples, Italy; ^2^UOSD di Fisiopatologia della Riproduzione, Presidio Ospedaliero di Marcianise, Caserta, Italy

**Keywords:** circRNAs, epigenetic signature, infertility, asthenozoospermia, mitochondria-dependent ceRNET

## Abstract

The role of circRNA in reproduction is under investigation. CircRNAs are expressed in human testis, spermatozoa (SPZ), and seminal plasma. Their involvement in embryo development has also been suggested. Asthenozoospermia, a common cause of male infertility, is characterized by reduced or absent sperm motility in fresh ejaculate. While abnormal mitochondrial function, altered sperm tail, and genomic causes have been deeply investigated, the epigenetic signature of asthenozoospermic derived SPZ still remains unexplored. CircRNAs may take part in the repertoire of differentially expressed molecules in infertile men. Considering this background, we carried out a circRNA microarray, identifying a total of 9,138 transcripts, 22% of them novel based and 83.5% with an exonic structure. Using KEGG analysis, we evaluated the circRNA contribution in pathways related to mitochondrial function and sperm motility. In order to discriminate circRNAs with a differential expression in SPZ with differential morphological parameters, we separated sperm cells by Percoll gradient and analyzed their differential circRNA payload. A bioinformatic approach was then utilized to build a circRNA/miRNA/mRNA network. With the aim to demonstrate a dynamic contribution of circRNAs to the sperm epigenetic signature, we verified their modulation as a consequence of an oral amino acid supplementation, efficacious in improving SPZ motility.

## Introduction

Spermiogenesis is the differentiation phase at the end of spermatogenesis that allows the transformation of round spermatids into spematozoa (SPZ), highly specialized cells provided with a head containing an elongated and transcriptionally inactive nucleus, an acrosome, and a tail, surrounded by a mitochondrial sheet at its midpiece (1, 2). Such a morphology is acquired through impressive remodeling events consisting of: (i) acrosome formation starting from Golgi-derived vesicles; (ii) histone replacement with protamines in order to obtain a tightly packaged chromatin; and (iii) global reorganization of the cytoskeleton architecture necessary for flagellum development (3). Sperm surely requires these changes to shield their own DNA during its journey along the epididymis and female reproductive tract, but, interestingly, all these changes also contribute to sperm quality and to the developmental program of the future embryo.

Low sperm count, abnormal morphology, and poor sperm motility are common causes of male infertility: according to the reports released by the World Health Organization (4), asthenozoospermia is defined as the total motility <40% and the progressive motility <32% in semen samples. Sperm motility is under the control of flagellum, a microtubule-based organelle able to convert chemical into mechanical energy, through glycolysis and oxidative phosphorylation, two metabolic pathways producing ATP, with the active participation of mitochondria (5, 6).

Mature sperm cells contain several types of RNAs with a potential key role in the oocyte upon fertilization (7, 8). Using a microarray strategy, the mRNA fingerprint has been drawn in fertile men (9) as well as in asthenozoospermic patients (10). This strategy has been deeply applied to unveil several other classes of non-coding RNAs (11). Among them, microRNAs (miRNAs) have an altered expression in semen, sperm, and testicular biopsy samples of infertile men (12, 13). Long non-coding RNAs show a similar behavior, changing their expression as a consequence of altered sperm functions (14). Piwi-interacting RNAs (piRNAs) take part to differentially expressed non-coding RNAs in the seminal plasma of infertile men (15). Studies that focus on the identification of differentially expressed RNAs in infertile patients, when compared to fertile controls, may shed light on the pathogenic mechanisms involved in asthenozoospermia.

Until today, the characterization of a circular RNA (circRNA) cargo in SPZ, collected from infertile men, has never been carried out. The role of circRNA has been deeply investigated in both pathological and physiological conditions (8, 16). With regard to reproductive organs, circRNAs has been identified in both testis and ovary (17, 18) and their distribution has been correlated to germ cell progression (19). Human SPZ also retain circRNAs; interestingly, these molecules are differentially expressed in populations of SPZ with good and poor quality and show a peculiar subcellular localization in SPZ head and tail-enriched preparations to indicate that they are preserved during SPZ maturation and transferred into oocyte during fertilization (20, 21). In support of this hypothesis, a particular circRNA (circNAPEPLDiso1) has been found and functionally characterized in both human and mouse SPZ: in detail, it physically interacts with miRNAs, especially involved in the control of the cell cycle, and its expression increases in murine fertilized oocytes as a consequence of a paternal cytoplasmic contribution to the zygote (21).

Although the experimental design detailed here partially overlaps with that used in Chioccarelli et al. (20), in the present paper we demonstrate—for the first time—the circRNA payload in SPZ collected from infertile men. SPZ from asthenozoospermic patients retain, in fact, circRNAs in a total number of 9,138, identified using a microarray strategy. A differential analysis was carried out in two populations of SPZ (A SPZ= good quality, B SPZ = low quality) separated by Percoll gradient on the basis of morphology and motility parameters, in order to identify differentially expressed (DE)-circRNAs.

Thinking at circRNAs as potential biomarkers of sperm quality, beyond classical morphological parameters, we evaluated their modulation in SPZ of asthenozoospermic patients before and after pharmacological treatments aimed at improving their clinical profile. Interestingly, the expression of selected circRNAs—previously identified as biomarkers of A SPZ in normozoospermic volunteers (20)—significantly decreased and increased in pre- and post-treated asthenozoospermic patients, respectively. On the other hand, the expression of selected circRNAs, here identified as biomarkers of A SPZ in asthenozoospermic patients, significantly decreased after pharmacological treatment.

Despite that, the molecular mechanism that underlies such a modulation is still under investigation. The data shown here enrich the picture concerning circRNAs and point to these molecules as being involved in signaling pathways linked to asthenozoospermia.

## Materials and Methods

### Human Semen Samples

Semen samples of asthenozoospermic (*n* = 30) and normozoospermic (*n* = 10) patients were obtained from the Marcianise Hospital Unit-UOSD of Physiopathology of Reproduction. Although most experiments were performed on asthenozoospermic patients, the cohort of normozoospermic volunteers was just analyzed as the experimental control in the pharmacological treatment, as described below. The age of all patients ranged from 27 to 39. Table 1 contains all the evaluated parameters in asthenozoospermic patients. After 3–5 days of sexual abstinence, semen samples were produced by masturbation and collected in sterile sample containers, which were delivered to the laboratory within 1 h after ejaculation. The sperm samples were allowed to liquefy for 30 min at 37°C before analysis by trained andrology staff. Semen parameters, such as volume, concentration, total motility, progressive motility, and morphology were assessed according to WHO (4) reference criteria using computer-assisted sperm analysis (CASA) technology. Sperm samples were then purified on a Percoll density gradient.

**Table 1:** Parameters evaluated in asthenozoospermic patients.

**Parameters evaluated**	**Asthenozoospermic patients**
Age of patients	33.14 ± 6.8
Semen total volume (ml)	2.17 ± 0.57
Sperm concentration (×10^6^/ml)	33.54 ± 12.31
Total motility (%)	33.59 ± 3.47
Progressive motility (%)	23.74 ± 4.94
Sperm vitality (%)	44.38 ± 8.39
Normal morphology (%)	14.6 ± 4.2

### Ethical Approval

Sperm collection was performed after obtaining written informed consent from all participants, both asthenozoospermic patients and normozoospermic volunteers, in accordance with the Declaration of Helsinki. Subjects were interviewed to better understand their area of origin, their eating habits, as well as their lifestyles. This study involving human participants was reviewed and approved by the ethics committee of Azienda Sanitaria Locale (ASL) Caserta, Regione Campania (n. 1353 del 27. 10. 2017).

### Asthenozoospermic Patient Treatment

The treatment of asthenozoospermic patients was carried out by administrating a mixture of amino acids, ornithine-citrulline-L-arginine, at a dose equal to 1,000 mg of one capsule per day for 3 months. The treatment was well-tolerated in all subjects. At the end of the treatment period, semen parameters were assessed and the percentage of motile SPZ significantly increased from 20 to 40%. In addition, SPZ were purified by Percoll gradient and A and B SPZ were obtained as described below.

### SPZ Isolation by Percoll Density Gradient Centrifugation

Purification of human SPZ was achieved using a 40 and 80% discontinuous Percoll (GE Healthcare, Castle Hill, Australia) centrifugation gradient. For this procedure, Percoll (90 ml) was supplemented with a 10 ml Dulbecco phosphate buffered saline (PBS) 10-fold concentrated solution (Lonza, Basel, Switzerland). The resulting solution (considered to be 100% Percoll) was further diluted with PBS 1x to give 40 and 80% Percoll solutions. The pH was equilibrated to 7.4. The gradient was prepared by placing 1 ml of each 40/80% solution in a conical plastic tube 30 mm in diameter. The human semen sample (1 ml) was loaded at the top of the gradient and centrifuged at 300 × *g* for 20 min. Following centrifugation, the seminal plasma was removed and discarded and each fraction was collected by aspiration, starting from the upper layer. In particular, purified viable and motile SPZ were recovered from the base of the 80% Percoll fraction (“A SPZ”) while abnormal SPZ were recovered from 40% Percoll fraction (“B SPZ”). A and B SPZ pellets were washed once with 10 ml of PBS to remove the Percoll, followed by centrifugation at 500 × *g* for 15 min, and analyzed in terms of motility and morphology using CASA technology. To do this, we took advantage of Sperm Class Analyzer (SCA) software that records the number of motile/immotile SPZ, with an accurate counting, and determines SPZ trajectory and velocity. SPZ morphology was, instead, evaluated by staining an aliquot of A and B pellets with SpermBlue, following the manufacturer's instructions.

In addition, both SPZ fractions were evaluated under light microscopy to estimate possible contaminations by somatic cells; thus, they were treated with Somatic Cell Lysis Buffer (SCLB) (9) consisting of 0.1% SDS, 0.5% Triton X-100 in DEPC-H_2_O. Lysis was proceeded by incubating cells on ice for 30 min. Microscope examination was used to verify the elimination of somatic cells. If somatic cells persisted, SCLB treatment was repeated by centrifuging the sample and resuspending the pellet in fresh SCLB. Finally, when somatic cells were absent, the sample was centrifuged at 200 × *g* for 15 min at 4°C; SPZ, contained in the pellet, counted by CASA, and frozen at −80°C prior to processing for total RNA isolation.

### Total RNA Preparation

Total RNA was extracted from human SPZ using Trizol^®^ Reagent (Invitrogen Life Technologies, Paisley, UK) following the manufacturer's instructions. In brief, the sample was homogenized in Trizol Reagent (1 ml Trizol^®^ Reagent/5–10 × 10^6^ sperm cells); after homogenization, the sample was incubated for 5 min at 20°C to permit the complete dissociation of nucleoprotein complexes. Then 0.2 ml chloroform/ml Trizol^®^ Reagent was added, and the sample was centrifuged at 12,000 × *g* for 15 min at 4°C. The aqueous phase was transferred to a fresh tube and total RNA was precipitated by mixing with isopropyl alcohol (0.5 ml/ml Trizol^®^ Reagent) and 1 μl glycogen (20 mg/ml) to promote the precipitation of small size RNAs. After centrifugation at 12,000 × *g* for 10 min at 4°C, the RNA pellet was washed with 75% ethanol, centrifuged at 7,500 × *g* for 10 min at 4°C and dissolved in an appropriate volume of DEPC treated water. The quantity (ng/ml) and purity (260/280 and 260/230 ratios) of total RNAs were assessed with a NanoDrop 2000 spectrophotometer (Thermo, Waltham, MA, USA). To remove potential contamination of genomic DNA, RNA aliquots (10 μg) were treated with 2U DNase I (RNase-free DNase I, Ambion, Thermo Fisher Scientific, Massachusetts, USA) according to the manufacturer's recommendations. Finally, total RNA was digested with RNase R (Ribonuclease R, *E. coli*, Cat. No. RNR07250, Epicenter, Madison, Wisconsin, USA) 10 U enzyme/1 μg RNA, at 37°C for 10 min to remove linear RNAs and to enrich samples of circRNAs, followed by heat inactivation at 95°C for 3 min. The RNAs were then preserved at −80°C until the next step.

### CircRNA Microarray

The sample preparation and microarray hybridization were performed according to the Arraystar's standard protocols (Arraystar, Rockville, MD). Briefly, the enriched circRNAs were amplified and transcribed into fluorescent cRNA utilizing a random priming method according to Arraystar Super RNA Labeling protocol (Arraystar, Inc.). The labeled cRNAs were purified by RNeasy Mini Kit (Qiagen). The concentration and specific activity of the labeled cRNAs (pmol Cy3/μg cRNA) were measured by NanoDrop ND-1000. One microgram of each labeled cRNA was fragmented by adding 5 μl 10× Blocking Agent and 1 μl of 25× Fragmentation Buffer, then the mixture was heated at 60°C for 30 min, and finally, 25 μl 2× Hybridization buffer was added to dilute the labeled cRNA. Fifty microliter of hybridization solution was dispensed into the gasket slide and assembled to the circRNA expression microarray slide. The slides were incubated for 17 h at 65°C in an Agilent Hybridization Oven. The hybridized arrays were washed, fixed and scanned using the Agilent Scanner G2505C (Agilent Technologies, CA).

### Expression Profiling Data

Agilent Feature Extraction software (version 11.0.1.1) was adopted to analyze acquired array images. Quantile normalization of raw data and subsequent data processing were performed using the R software package. After quantile normalization of the raw data, low intensity filtering was performed, and the circRNAs where at least three out of six samples had flags in “P” or “M” (“All Targets Value”) were retained for further analyses.

### Differential Expression Analysis

Two groups of circRNAs were identified in A and in B SPZ, respectively, using the R software package and were conveniently compared by *t*-test to select differentially expressed (DE)- circRNAs, with *p* ≤ 0.05 and fold changes ≥ 1.5. Since there was a comparison between A and B SPZ, we used the text the expression “B compared to A SPZ” to indicate statistically significant DE-circRNAs as shown by volcano plot filtering. DE-circRNAs among samples were identified through Fold Change filtering. Hierarchical clustering was performed to show the distinguishable circRNAs expression pattern among samples.

### Functional Annotation for circRNA/miRNA and Target miRNA Interaction

The circRNA/miRNA interaction was predicted with Arraystar's miRNA target prediction software. Based on both TargetScan (22) and MiRanda online analytical software (23); such an analysis was performed for all DE-circRNAs. For functional annotation, all parental genes of the DE-circRNAs were subjected to KEGG (www.genome.jp/kegg) pathway enrichment analyses using DAVID Bioinformatics Resources 6.8 (david.ncifcrf.gov/home.jsp). The *p*-value was calculated using a hypergeometric test and corrected by Benjamini-Hochberg adjustment. We regarded the Fold Enrichment as the enrichment score that indicated the significance of correlation. Validated or predicted targets of miRNAs were retrieved by Diana TarBase 8.0 (http://www.microrna.gr/tarbase); circRNA/miRNA/Target network was built and visualized using the Bisogenet plug-in of Cytoscape (www.cytoscape.org).

### PCR Primer Design

Primers to validate and amplify selected circRNAs in human SPZ samples were designed through the online tool Primer-BLAST (http://www.ncbi.nlm.nih.gov/tools/primer-blast/). In order to make primers specific for the circular isoforms, we designed primers spanning the back-splicing junction. We also designed specific primers for the housekeeping gene used for normalization: *GAPDH* (glyceraldehyde 3-phosphate dehydrogenase). Primers for human genes are shown in Table 2.

**Table 2:** Primer sequences and annealing temperatures for circRNAs.

**Gene primers**	**Sequences 5′-3′**	**Tm (°C)**
*circMCC* S	CCGGAAGACAGCTGAGAACG	52
*circMCC* AS	TGAAGCTCTCTGACCTGGTGT	
*circPAPPA2* S	GCTGAACGACTTTGACGACG	52
*circPAPPA*2 AS	TCAAACACACCTCTTGGGTGA	
*circSLC25A26* S	TGAAGAGGGTATCCAAGGGTT	52
*circSLC25A26* AS	AATCAGGCAGGCAACCTCTC	
*circCANX* S	TGGTTAGATGATGAGCCTGAGT	52
*circCANX* AS	AGGGGCATCTTCATCCCAATC	
*circDDX17* S	GGCCCAATCATTTGGAGCAAG	52
*circDDX17* AS	AAACGCTCCCCAGGATTACC	
*circHDAC3* S	TGCAGACCTCCTGACCTATGA	52
*circHDAC3* AS	CGGGAAACACTGGGCTGCTA	
*circSIRT5* S	GTCTAGTGGTGGGCACTTCC	54
*circSIRT5* AS	CATTTCCATTTACTGAATCTGTTCG	
*circDYNC1H1* S	AACATCGACACGGTTGCTCT	54
*circDYNC1H1* AS	CCTGGGTGAATCTCTCCTTTGA	
*circFABP6* S	GGGAGGTGATGTGTGATTTGC	56
*circFABP*6 AS	AGGTATGCTCTCCCCTACACC	
*circTADA2A* S	GCAGAATGGGACTTGAGAGACAT	56
*circTADA2A* AS	GGGCCATTTCTTCTTGAGCA	
*circPEX1* S	CTCCATCTTGGGAAAGTCTGGG	56
*circPEX1* AS	AGCATGCAGCTCCAGTATCTC	
*circATF6* S	CACTTTCTCCAGCCTCCTCAA	56
*circATF6* AS	AGAGCAGAATAGGAACATGCTGA	
*circUSP54* S	CAATGAGCCAGGGCAAAACA	52
*circUSP54* AS	GTGTCAGAGAGCTTGAGAGC	
*circCLSPN* S	AGCAGCATGGGTGATCCAAT	52
*circCLSPN* AS	AACATTTAAGAACTTGTCTGTGGG	
*circTRMT2B* S	TCGAAACTTCAGGGCCATCC	52
*circTRMT2B* AS	AGGCCAATCACACTCAATGACA	
*circCIT* S	GCAGCGAGAGGAGTACTTGC	52
*circCIT* AS	TCCAAGCAGTTTCAAAGGCCA	
*circEPS15* S	CCTTTTGTTGGCAATCTCTTCTC	52
*circEPS15* AS	CGGCTCAGCTCTTCTCTAGC	
*circPTBP3* S	CTGCGCATTGACTTCTCCAA	52
*circPTBP3* AS	ATCAGATCCCCGCAAAAGCA	

### CircRNA Expression Analysis by One-Step Evagreen qRT-PCR

We investigated circRNA expression through One-Step Evagreen qRT-PCR reaction using a kit containing qRT-PCR enzyme mix and an Evagreen qPCR Mastermix (Applied Biological Materials Inc.), according to the manufacturer's instructions. All reactions were performed using 50 ng of total RNA on a CFX-96 Real Time PCR System (Biorad). Assays were carried out in triplicate and included a melting curve analysis for which all samples displayed single peaks for each primer pair. A negative control, without RNA, was also included. RNA expression was evaluated through CFX Manager software (Biorad); normalization was performed using *GAPDH* as the housekeeping gene. Normalized fold expression of circRNAs was calculated by applying the 2^−ΔΔCt^ method.

### Statistical Analysis

All the data are expressed as the mean ± SEM for triplicate independent measurements. Student's *t*-test was used to assess the differences between experimental groups. Differences with *p* < 0.05 were considered statistically significant.

## Results

### Overview of circRNA Expression in Human SPZ From Asthenozoospermic Patients

**Figure 1:**
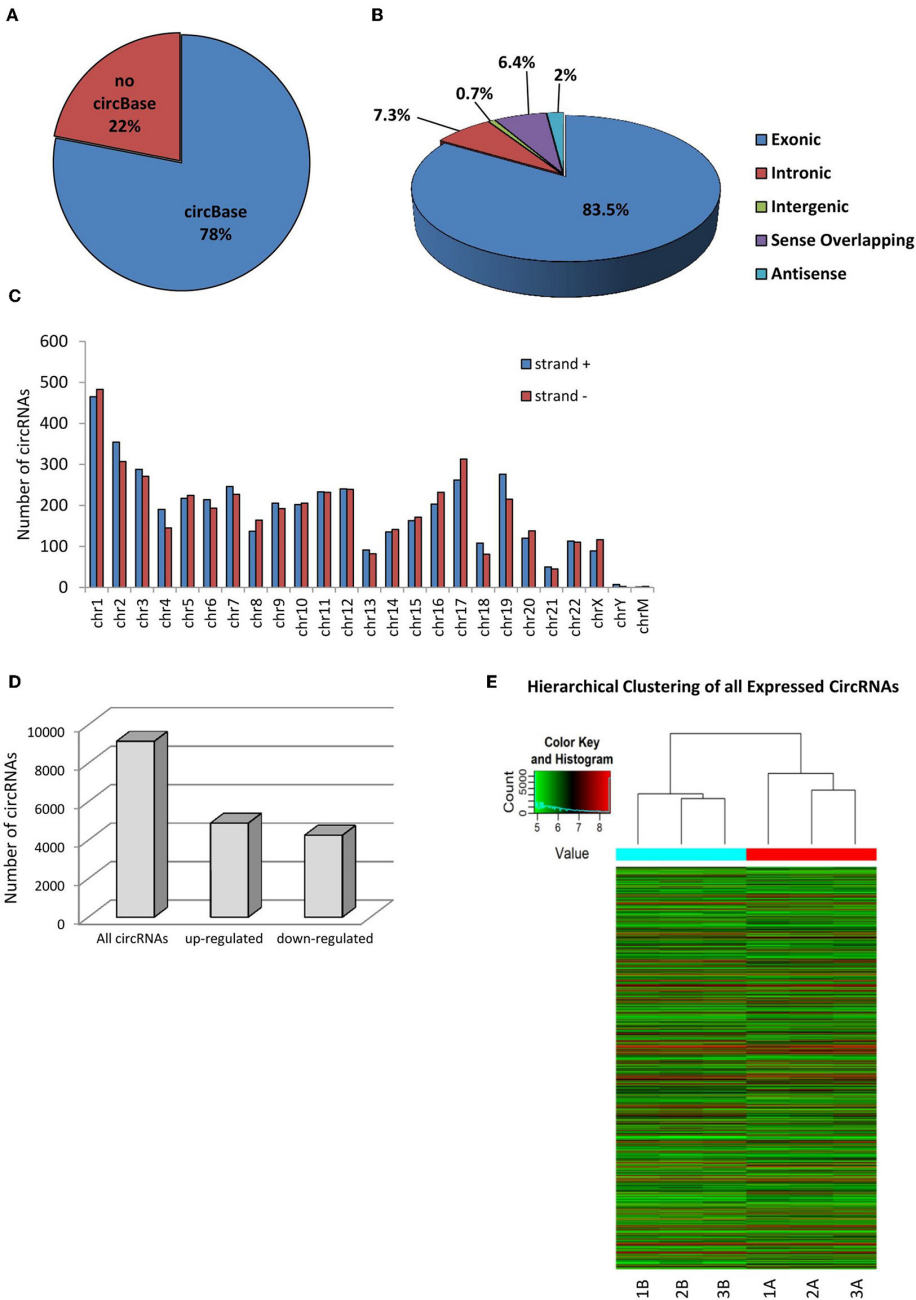
Overview of circRNA expression in asthenozoospermic derived SPZ. **(A)** The proportion of circRNAs from circBASE (http://circbase.mdc-berlin.de) and other databases/literature on a total of 9,138 circRNAs identified. **(B)** The proportion of different types of circRNAs among all predicted circRNAs. **(C)** Chromosomal distribution of SPZ derived circRNAs, on strand + and strand –. **(D)** The distribution of up- and down-regulated circRNAs in fraction B compared to fraction A asthenozoospermic derived SPZ among all circRNAs. **(E)** Hierarchical clustering analysis of total circRNAs samples arranged into two groups, based on their expression levels; in detail, this analysis used different colors to represent the expression values of circRNAs detected in fraction A (*n* = 3, indicated as 1A, 2A, 3A) and B (*n* = 3, indicated as 1B, 21B, 3B) SPZ of asthenozoospermic patients.

Human SPZ collected from asthenozoospermic patients (*n* = 3) were used to profile circRNAs using a microarray strategy; they retained a total of 9,138 circRNAs of which the majority (78%) are already present in the most common circRNA database (circBase), whereas 22% was novel based (Figure 1A). In accordance with their structure, circRNAs were distinguished in exonic, intronic, intergenic, sense overlapping, and antisense. The most abundant type of circRNAs in SPZ was the exonic one (83.5%), while intergenic (0.7%) and antisense (2%) were less represented (Figure 1B). CircRNAs were also analyzed on the basis of the location of their host genes: circRNAs were widely distributed across all chromosomes, including the mitochondrial (M) and Y chromosome, even if at a lesser extent, and on strand + or – (Figure 1C). Chromosomes 1–3, and 17 produced the highest number of circRNAs, whereas chromosomes Y and M generated the lowest number. Furthermore, circRNAs distributed on chromosomes 2–4, 6, 7, 9, and 19 predominantly derived from strand +; this profile was reversed for chromosomes 1, 8, 16, 17, 20, and X (Figure 1C). In order to investigate the possible involvement of the circRNA-dependent network (ceRNET) in asthenozoospermia, we separated SPZ collected from asthenozoospermic patients, in two different populations, on the basis of morphology and motility parameters, through a Percoll gradient separation (24). High quality SPZ with good morphology and motility were collected as a pellet on the bottom of 80% Percoll solution and was named A SPZ. We then collected, on the bottom of 40% Percoll solution, the fraction B containing SPZ of very poor in quality and which was not suitable for fertilization. Of the total number of circRNAs identified, *n* = 4,254 were circRNAs up-regulated in B compared to A SPZ, whereas *n* = 4,884 were down-regulated in B compared to A fraction (Figure 1D). A hierarchical clustering plot of all expressed circRNAs is also shown (Figure 1E).

### Identification and Functional Annotation of DE-circRNAs in Fraction B Compared to A Asthenozoospermic Derived SPZ

**Figure 2:**
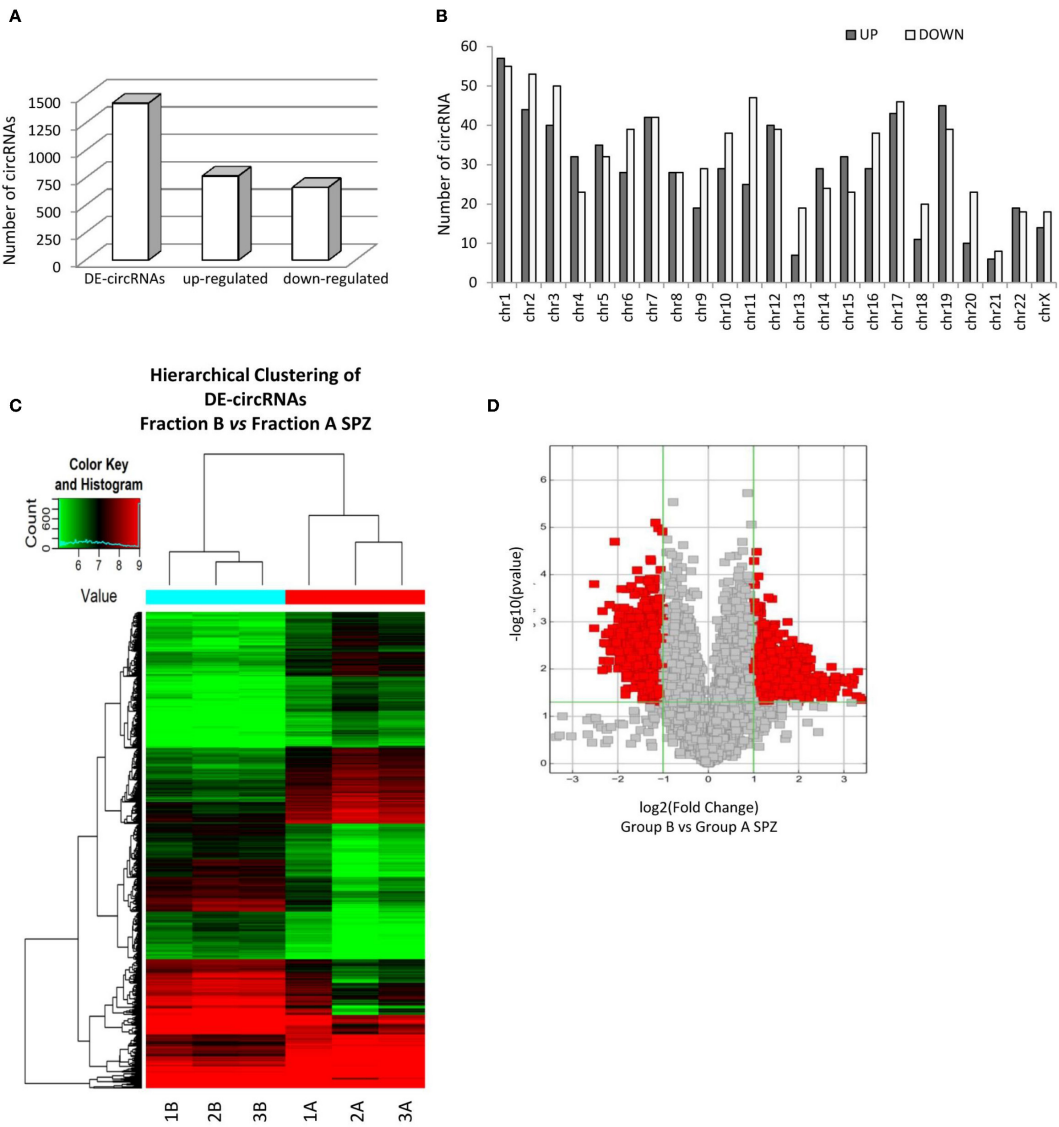
Differential expression of circRNAs between fraction A and B asthenozoospermic derived SPZ. Differential expression of circRNAs between fraction A and B SPZ. **(A)** The distribution of up- and down-regulated DE-circRNAs in fraction B compared to fraction A SPZ. **(B)** The distribution of up- and down-regulated DE-circRNAs in the human genome, according to their host gene location. **(C)** Hierarchical clustering analysis of DE-circRNAs in A SPZ (samples 1A, 2A, 3A) and B SPZ (samples 1B, 2B, 3B). The expression values (Fold change ≥ 1.5, *p* ≤ 0.05) are represented in different colors, indicating expression levels above and below the median expression level across all samples. **(D)** The volcano plot was constructed using Fold-Change and *p*-values; in detail, the values on X and Y axes are log2 (FC = Fold-Change) and –log10 (*p*-values), respectively. Red points in the volcano plot represent the DE-circRNAs with statistical significance.

By using more stringent filters, such as fold change cut-off ≥ 1.5 and *p*-values cut-off ≤ 0.05, circRNA microarray analysis identified a total of 1,432 DE-circRNAs in fraction A (*n* = 3) and fraction B (*n* = 3) SPZ, consisting of 664 up-regulated and 768 down-regulated circRNAs in B compared to A SPZ, respectively (Figure 2A). The distribution of DE-circRNAs in accordance to their host gene location was analyzed in the human genome (Figure 2B); interestingly, chromosomes 1, 4, 5, 12, 14, 15, 19, and 22 in particular contained up-regulated circRNAs in B compared to A SPZ; such a profile was reversed on chromosomes 2, 3, 6, 9–11, 13, 16–18, 20, 21, and X. No significant difference was observed on chromosomes 7, 8, and 22; no DE-circRNA was detected on chromosomes Y or M (Figure 2B). DE-circRNAs were clustered based on their expression levels in B and A SPZ, as indicated by the hierarchical clustering plot (Figure 2C) and the volcano plot, the last one representing DE-circRNAs as red points (Figure 2D).

**Figure 3:**
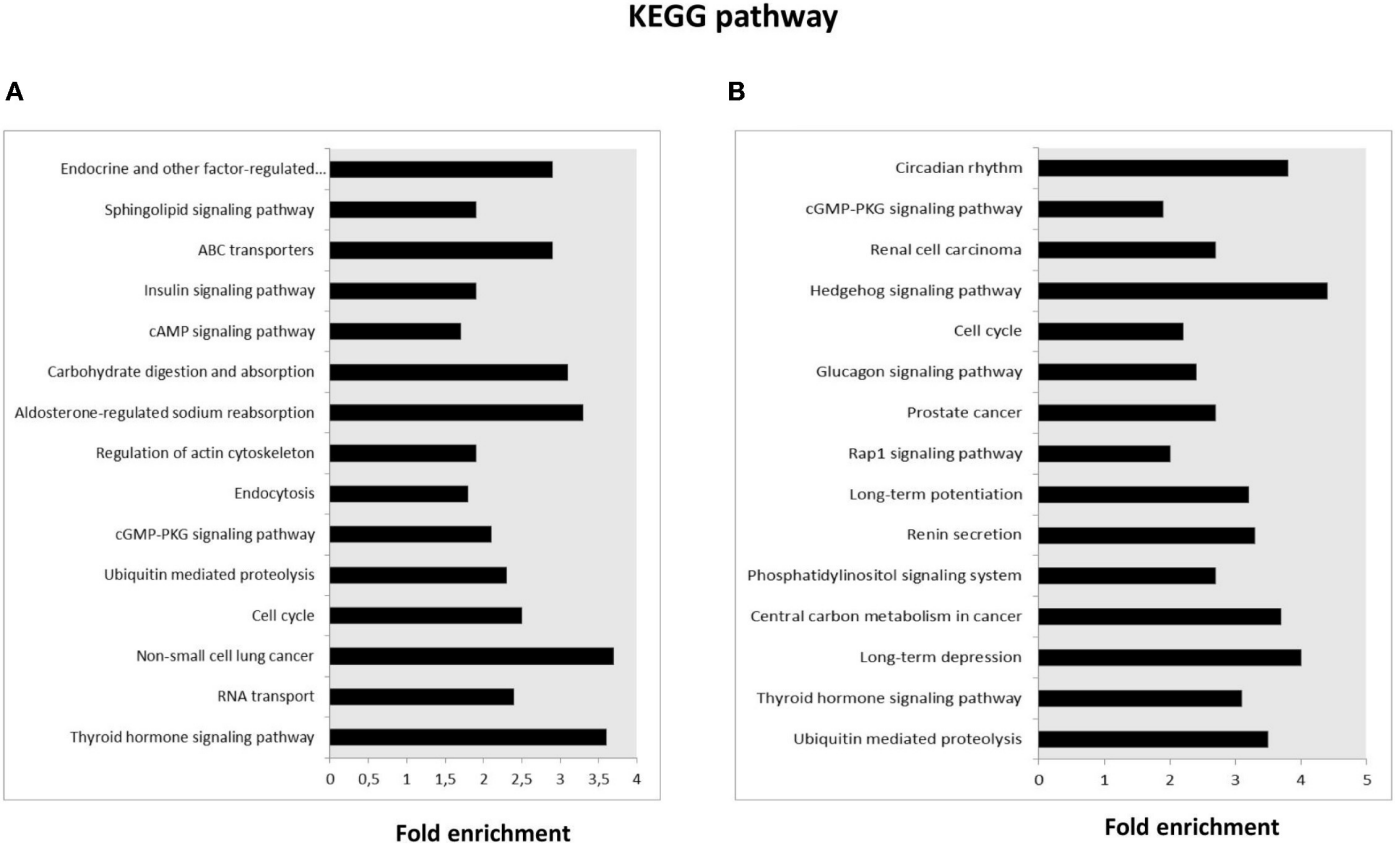
Kyoto Encyclopedia of Genes and Genomes (KEGG) pathway annotation of host genes. **(A)** The Top 15 KEGG signaling pathway annotations of circRNAs up-regulated in fraction A asthenozoospermic derived SPZ. **(B)** The Top 15 KEGG signaling pathway annotations of circRNAs down-regulated in fraction A asthenozoospermic derived SPZ.

KEGG pathway analysis was then performed for the target genes of SPZ-derived circRNAs; the Top 15 enriched KEGG signaling pathways are shown in Figures 3A,B. Interestingly, the KEGG pathways associated with up-regulated circRNAs in A SPZ were linked to sphingolipid signaling, cAMP signaling, insulin signaling, ABC transporters, aldosterone-regulated sodium reabsorption, carbohydrate digestion, and absorption (Figure 3A). The KEGG pathways associated with up-regulated circRNAs in B SPZ were linked to long-term depression and potentiation, central carbon metabolism in cancer, phosphatidylinositol signaling system, renin secretion, Rap1 signaling, cell cycle, Hedgehog signaling, and the circadian rhythm (Figure 3B).

### Experimental Validation of Predicted circRNAs in Asthenozoospermic Derived SPZ

Experimental validation of circRNA microarray results was carried out. In particular, a quality control of RNA extracted from SPZ was conducted as previously described (20).

**Figure 4:**
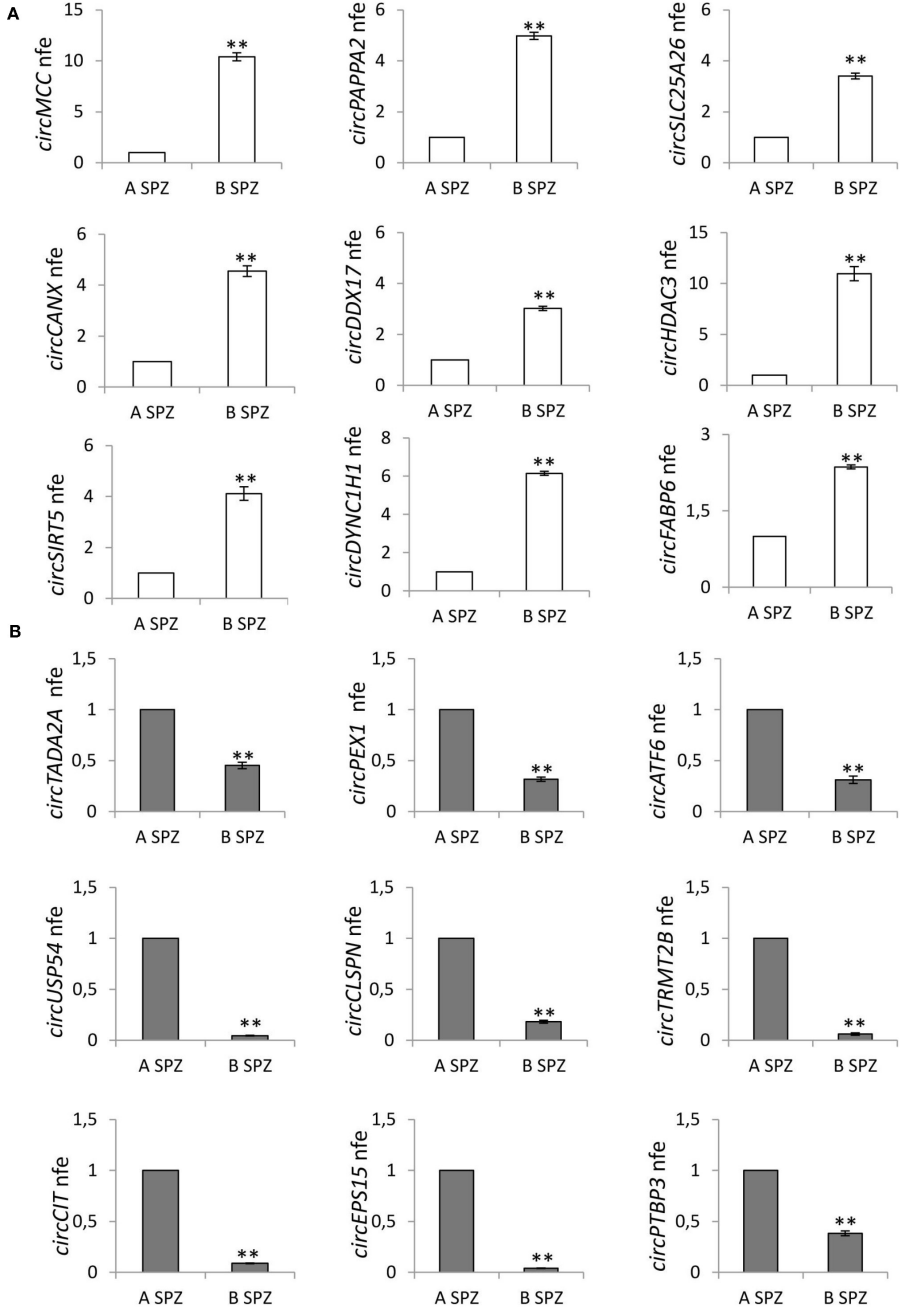
**(A)** Validation of the circRNA-microarray results up-regulated in B compared to A asthenozoospermic derived SPZ; ^**^p < 0.01. **(B)** Validation of the circRNA microarray results down-regulated in B compared to A asthenozoospermic derived SPZ; ^**^p < 0.01. A significant high score of normalized intensity and related host genes involved in sperm physiology and embryonic development functions represent the “selection criterion” of the DE-circRNAs validated.

For the validation by qPCR, the choice of circRNAs—predicted in asthenozoospermic derived SPZ and identified by circRNA microarray—was not random, rather, we used a selective functional criterion: we chose DE-circRNAs with a significant high score of normalized intensity and whose host genes were related to sperm physiology and embryonic development functions. Thus, we searched relative sequences in circBase and designed specific primers for circular isoforms, spanning the back-splicing junction to use for One-Step Evagreen qRT-PCR analysis (Figures 4A,B). The quality of melting curves for each primer pair was carefully checked and only curves with single peaks were considered suitable for further analysis.

qPCR analysis performed in A (*n* = 15) and B SPZ (*n* = 15) showed the expression of circRNAs up-regulated (*circMCC, circPAPPA2, circSLC25A26, circCANX, circDDX17, circHDAC3, circSIRT5, circDYNC1H1, circFABP6*; *p* < 0.01; Figure 4A) and down-regulated (*circTADA2A, circPEX1, circATF, circUSP54, circCLSPN, circTRMT2B, circCIT, circEPS15, circPTBP3*; *p* < 0.01; Figure 4B) in B compared to A SPZ, respectively, thus, confirming circRNA microarray results.

### Construction of a *circUSP54* -Dependent ceRNET

**Figure 5:**
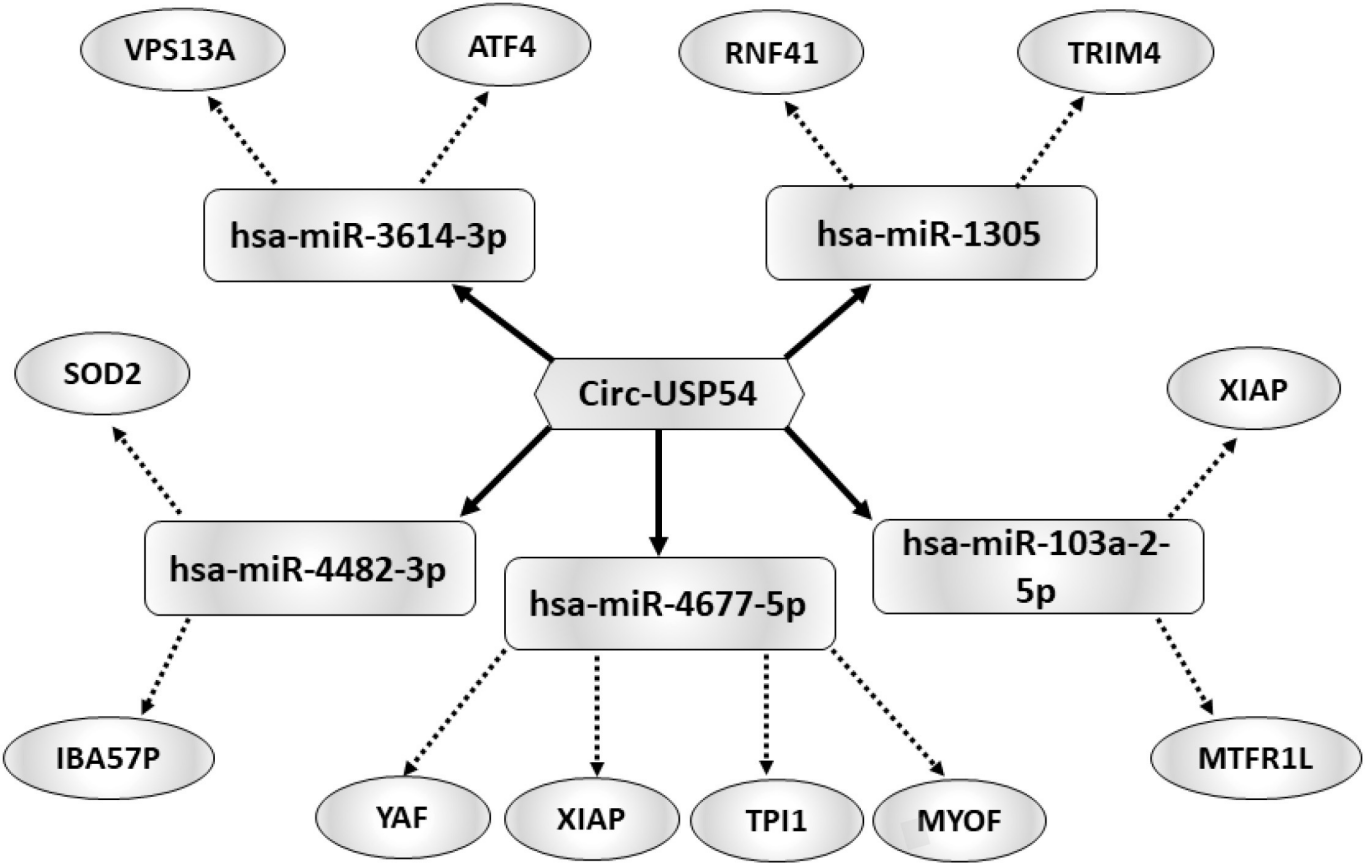
Functional clustering of DE-circRNA down-regulated in B compared to A asthenozoospermic derived SPZ. One circRNA, *circUSP54*, tethers a group of miRNAs as targets, all involved in mitochondria-dependent pathways. Networks were built using Cytoscape. Hexagonal and rectangular symbols represent circRNAs and miRNAs, respectively. The arrow indicates the tethering activity of circRNAs toward miRNAs, while the dotted arrow indicates the pathways upstream of the miRNAs.

Considering that circRNAs are able to harbor several miRNAs (25), the construction of a ceRNET is useful to shed light on predicted mRNA targets. Among circRNAs up-regulated in A SPZ of asthenozoospermic patients, we were interested in *circUSP54* since its linear transcript encodes for a ubiquitin-specific processing protease (USP). USPs are deubiquitinating enzymes with key roles in mitochondrial morphology and, in general, in the control of fertility (26, 27). According to the results of bioinformatic prediction (Figure 5), five miRNAs were identified: has-miR-3614-3p, has-miR-1305, has- miR-103a-2-5p, has-miR-4677-5p, and has-miR-4482-3p. mRNAs targets were preferentially involved in mitochondria physiology as discussed below.

### Expression of DE-circRNAs in Fraction A SPZ of Asthenozoospermic Patients After Oral Amino Acid Supplement

**Figure 6:**
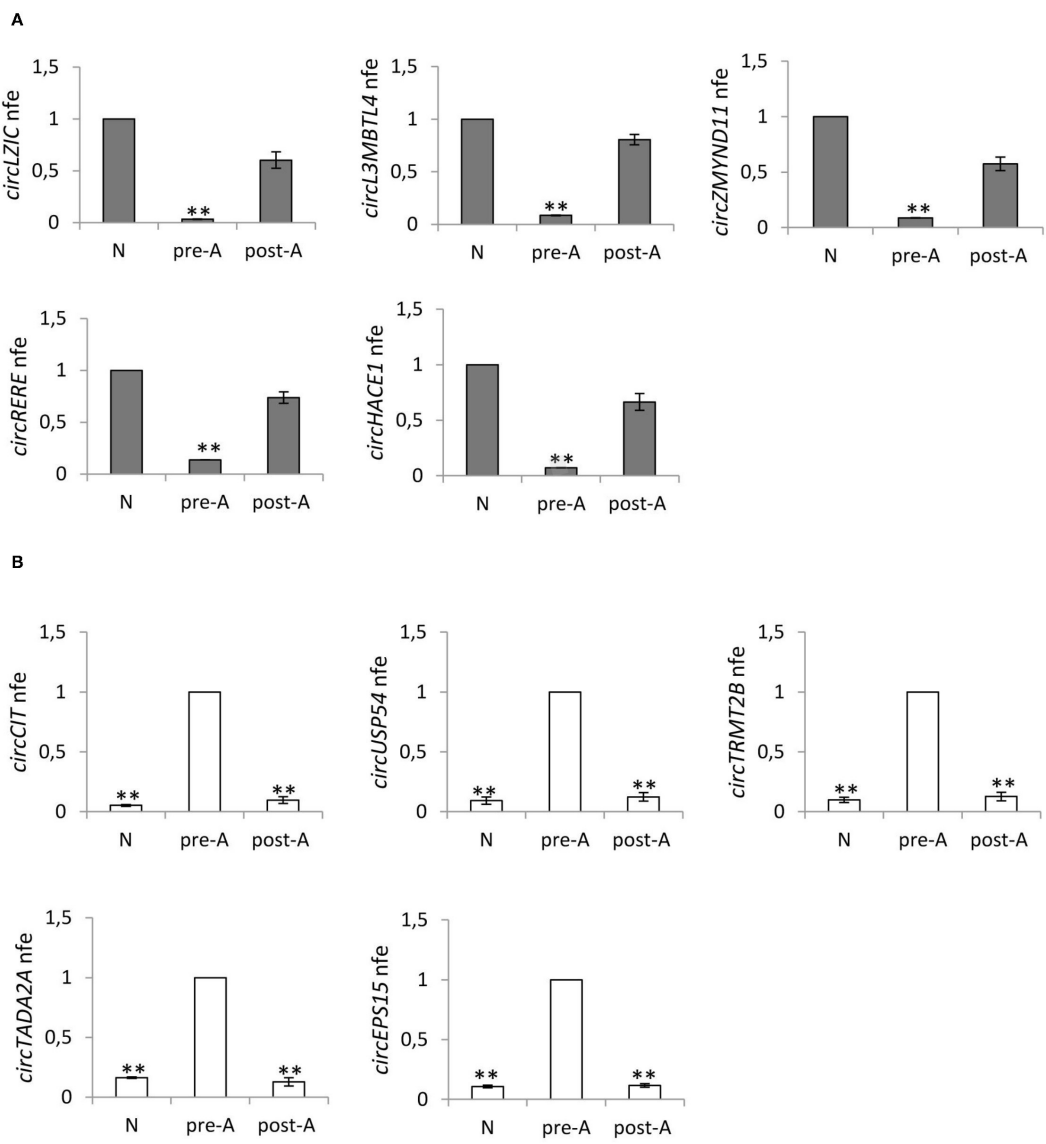
**(A)** Expression of five circRNAs up regulated in A normozoospermic derived SPZ (N) compared to A asthenozoospermic derived SPZ from men pre- (pre-A) and post-treatment with oral amino acid supplement (post-A); ^**^p < 0.01. **(B)** Expression of five circRNAs up-regulated in A asthenozoospermic derived SPZ from men pre-treatment with oral amino acids supplement (pre-A) compared to A normozoospermic derived SPZ (N) and to A asthenozoospermic derived SPZ from men post-treatment with oral amino acids supplement (post-A); ^**^p < 0.01.

With the aim of testing the effects of the oral amino acid supplement on circRNA content in SPZ from asthenozoospermic patients in pre- (*n* = 12) and post- (*n* = 12) treatment, we analyzed the expression of five circRNAs up-regulated in A SPZ of normozoospermic volunteers (Figure 6A) and five circRNAs up-regulated in A SPZ of asthenozoospermic patients (Figure 6B). In detail, the circRNAs up-regulated in A SPZ of normozoospermic volunteers were chosen from our previous work (20), among those preferentially localized in the sperm head and, therefore, considered potential markers of sperm quality to be transmitted to the oocyte during fertilization. Their expression was then evaluated in all three experimental groups: SPZ of normozoospermic volunteers (N), SPZ of pre-treated asthenozoospermic patients (pre-A), and SPZ of post-treated asthenozoospermic patients (post-A), to verify if the pharmacological treatment may allow sperm of asthenozoospermic patients to recover an epigenetic signature similar to SPZ of normozoospermic volunteers.

Figure 6A confirms our hypothesis, showing that the expression of circRNAs—up-regulated in (N)—significantly decreased in pre-A (*p* < 0.01) and was completely reverted in post-A (*p* < 0.01). In addition, we also chose circRNAs up-regulated in A SPZ of asthenozoospermic to verify whether after the pharmacological treatment, their expression decreased at levels of N. Figure 6B confirms our hypothesis, showing that the expression of circRNAs up-regulated in pre-A, significantly decreased in N, and was also very low in post-A.

## Discussion

Asthenozoospermia—characterized by impaired sperm motility—is an important reason for male infertility. Currently, several molecular mechanisms have been explored to better understand the phenomenon.

The integrity of the sperm tail structure is under the control of more than 1,000 proteins, as suggested by proteomic studies, most of them related to metabolism, especially the lipid metabolism, and energy production (28, 29). Interestingly, their post-translational modifications, mainly phosphorylation, follow a differential profile between normozoospermic and asthenozoospermic subjects (30). The possibility of causative genes for asthenozoospermia is also very strong; therefore, knockout mouse models, with an affected sperm tail structure, are useful to disentangle the molecular network involved (31, 32). The molecular motor for flagella movement is adenosine triphosphate (ATP), produced by two metabolic pathways in different regions of the flagellum: glycolysis and oxidative phosphorylation. Thus, sperm motility is dependent on the integrity of the mitochondria (33, 34). In fact, structural and functional alterations in mitochondria from asthenozoospermic subjects confirm the role of these organelles in energy maintenance (35).

Beyond haploid nuclear genome, SPZ are important vectors of the epigenetic signature for the zygote, deemed crucial for normal embryo development (8, 36). The sperm nucleus needs to reduce the size by packing its genome, through the replacement of histones with protamines (37, 38). Paternal DNA also undergoes a global methylation that serves to shut down the genome (39). Beyond DNA methylation, histone-to-protamine replacement and histone post-translational modifications, a large cargo of RNAs enriches SPZ. SPZ-borne RNAs constitute a heterogeneous family of both coding and non-coding RNAs (40). Sperm epigenetic signature significantly changes in male infertility cases (10, 41–45). Using a microarray-based strategy, the mRNA fingerprint has been characterized in fertile men (9). SPZ also contain a subset of sperm RNAs, involved in the control of sperm physiology, fertilization, and embryo development, differentially expressed in fertile and infertile subjects (11, 46, 47). These changing profiles suggest their potential of acting as markers for fertility evaluation. Accordingly, mRNA and lncRNA transcriptomes have been provided in bull semen, suggesting the influence of these RNAs on sperm motility (48).

In this scenario, the contribution of circRNAs in the pathogenic mechanisms at the base of asthenozoospermia is a novel aspect investigated here. With this in mind, we first identified a complete profiling of 9,138 circRNAs in asthenozoospermic derived SPZ, and 22% of them were novel based. Albeit with the same experimental approach used in Chioccarelli et al. (20), our purpose was to identify the circRNA payload in SPZ collected from infertile men, adding a new piece of knowledge to what has already been discussed for normozoospermic subjects (20). As in human testis, in ovary and normozoospermic derived SPZ (17, 18, 20), exonic circRNAs were the highest number and were widely scattered across all chromosomes, sexual and mitochondrial chromosomes included. In order to identify a differential cargo of circRNAs in correlation to sperm quality, we separated SPZ that have a good morphology and motility (A SPZ) from SPZ that is not suitable for fertilization (B SPZ). Interestingly, circRNA microarray analysis identified a total of 1,432 DE-circRNAs in fraction A and fraction B SPZ, consisting of 664 up-regulated and 768 down-regulated circRNAs in B compared to A SPZ. This result immediately peaked our attention considering that in normozoospermic derived SPZ we distinguished just 148 DE-circRNAs, using exactly the same experimental approach (20). It is plausible that sperm with altered motility has its own circRNA payload. This result is intriguing and poses new questions about the real effectiveness of the intracytoplasmic sperm injection (ICSI) procedure which has been considered a useful approach to bypass motility defects and to select good quality SPZ for fertilization, even in asthenozoospermic patients (49). Data shown here clearly suggest that good quality SPZ, isolated using standard procedures on the basis of morphological parameters, vary between normozoospermic and asthenozoospermic subjects, since they have a differential quantitative and qualitative fingerprint of circRNAs, therefore, they are cells with unique epigenetic signature.

CircRNA profiling has also been validated selecting circRNAs with a significant high score of normalized intensity and whose host genes were related to sperm physiology and embryonic development function. Such an analysis perfectly mirrored the circRNA microarray results. Using KEGG annotation, we discovered several important pathways related to energy metabolism and, therefore, likely related to sperm motility, involving DE-circRNAs. Interestingly, circRNAs in normozoospermic derived SPZ were, in particular, linked to DNA duplication, cell cycle, and oocyte meiosis, which are biological processes important for the first stages of embryo development (20). Conversely, DE-circRNAs analyzed here have been found to be involved in cAMP and insulin signaling, ABC transporters, long-term depression and potentiation, the phosphatidylinositol signaling system, renin secretion, Rap1 signaling and Hedgehog signaling, and the circadian rhythm which are important enriched pathways that are related to mitochondria function and sperm motility (50–53). The construction of a ceRNET is a useful instrument to shed light on predicted mRNA targets of circRNAs (25). Interestingly, we focused our attention on a representative circRNA, up-regulated in A SPZ of asthenozoospermic patients, *circUSP54*, to evaluate its interaction network. USPs are well-known deubiquitinating enzymes with key roles in spermatogenesis: *USP9* deletion has been discovered in infertile men (54), polymorphisms in *USP26* have been associated with infertility (55), male mice lacking *USP2* have severe defects in sperm motility (56), and USP30 regulates mitochondrial morphology (26). Both SPZ and seminal plasma have a specific miRNA signature that differentially changes in infertile men (57). *CircUSP54—*through has-miR-4482-3p—controls the expression of both *SOD2* and *IBA57P*, an enzyme that converts superoxide to less reactive hydrogen peroxide (58) and a protein located in the mitochondrial matrix, essential for mitochondrial DNA maintenance (59), respectively. Several other mRNA targets downstream of *circUSP54* are involved in mitochondrial activity, such as *VPS13A* (60), *XIAP* (61), *MYOF* (62), and *TRIM4* (63). Some mRNA targets also have key roles in semen quality such as in the case of *SOD2* (64) and *XIAP* (65); more intriguingly is the role of *VPS13A*, whose loss-of-function causes an abnormal ultrastructural morphology of the mitochondria located in the sperm midpiece, with dramatic effects on sperm motility (66). In addition to recent discoveries of circRNAs in human normozospermic (20) and asthenozoospermic (present data) SPZ, we also evaluated the possibility that the circRNA pattern may fluctuate with a high degree of plasticity as a consequence of a pharmacological treatment. This a novel aspect that may contribute to the view of the potential activity of circRNAs as biomarkers. With this in mind we verified the effect of an oral amino acid supplementation—with consolidated action on vitality and motility of SPZ (67–69) on the expression pattern of selected circRNAs. Interestingly, our analysis highlighted that circRNAs, such as *circLZIC, circL3MBTL4, circZMYND11, circRERE*, and *circHACE1*, up-regulated in A SPZ of normozoospermic volunteers (20), showed low and high expression in asthenozoospermic patients pre- and post- treatment, respectively. Conversely, circRNAs identified as potential markers of A SPZ of asthenozoospermic patients, such as *circCIT, circUSP54, circTRMT2B, circTADA2A*, and *circEPS15*, that had low levels of expression in normozoospermic derived SPZ, inverted their profile in asthenozoospermic derived SPZ after oral amino acid supplementation. These data clearly suggest that circRNA payload carried by SPZ is adjustable and dynamically changes in relation to sperm motility. Although the advances in etiology of male infertility and several cellular and molecular factors have been identified to be involved in the onset of asthenozoospermia, molecular mechanisms impairing sperm motility requires a deeper investigation. Accordingly, ideal therapies for such a disease have not been established. CircRNAs molecules may be involved and their modulation downstream oral amino acid supplementation is known to improve sperm vitality and motility, inspiring confidence. However, more effort is required to better understand the enzymatic pathways related to circRNA biogenesis in sperm cells.

## Data Availability Statement

The dataset for this manuscript are not publicly available with respect for individual privacy of participants. Requests to access the datasets should be directed to Dr. Rosanna Chianese, rosannachianese@unicampania.it.

## Ethics Statement

The studies involving human participants were reviewed and approved by Regione Campania - acting as Azienda Sanitaria Locale (ASL) Caserta—prior agreement of ethics committee (n. 1353 del 27. 10. 2017). The patients/participants provided their written informed consent to participate in this study.

## Author Contributions

RC, TC, and FM: conceptualization. GC, RC, and TC: methodology. BF, CS, GM, and FM: validation. TC and RC: writing—original draft preparation and writing—review and editing. TC and FM: figure preparation. SF and GC: visualization. RC and RP: supervision. RP: funding acquisition. All authors: contributed to the article and approved the submitted version.

## Conflict of Interest

The authors declare that the research was conducted in the absence of any commercial or financial relationships that could be construed as a potential conflict of interest.
